# Matrix metalloproteinase-2 (*MMP2*) rs243865 polymorphism and target end-organ damage in difficult-to-control hypertensive patients

**DOI:** 10.7717/peerj.20489

**Published:** 2026-03-06

**Authors:** Thuc Tri Nguyen, An Viet Tran, An Tuan Huynh, Quyen Thuy Nguyen, Linh Giao Ly Pham, Bao The Nguyen

**Affiliations:** 1 Cho Ray Hospital, Ho Chi Minh City, Vietnam; 2 Can Tho University of Medicine and Pharmacy, Can Tho City, Vietnam

**Keywords:** MMP-2 rs243865 polymorphism, Difficult-to-control hypertension, Target organ damage

## Abstract

**Background:**

The matrix metalloproteinase-2 (*MMP2*) rs243865 polymorphism has been associated with cardiovascular events; however, its impact on difficult-to-control hypertension remains unclear. This study aims to assess the characteristics of rs243865 polymorphism and its association with target organ damage in adult patients with difficult-to-control hypertension in Vietnam.

**Methods:**

A cross-sectional study was conducted on 70 difficult-to-control hypertensive patients at two medical centers in Southern Vietnam. All patients underwent Sanger sequencing for analysis of the *MMP2* rs243865 polymorphism. Target organ damage (TOD) was assessed across cardiac (left ventricular hypertrophy (LVH), myocardial ischemia), renal (decreased glomerular filtration rate, microalbuminuria), and vascular (carotid stenosis, peripheral vascular disease) systems, with patient-level counts of damaged target-organ systems derived. Data were processed in R 4.5.0 (RStudio, 2025), and associations were analyzed using logistic regression with false discovery rate (FDR) control based on Storey’s q-values.

**Results:**

Analysis of the *MMP2* rs243865 polymorphism revealed a predominance of the C allele (87.1%), with the CC genotype being the most common (75.7%). Allele/genotype distributions did not differ across clinical features (*p* > 0.05) except for higher dyslipidemia in C-allele carriers and the CC genotype (*p* = 0.035 and *p* = 0.033). Adjusted models (age, sex, dyslipidemia, duration of hypertension, and duration of diabetes) showed the C allele and CC genotype associated with microalbuminuria (*q* = 0.041; *q* = 0.049), echocardiographic LVH (*q* = 0.039; *q* = 0.027), and renal TOD (*q* = 0.019; *q* = 0.027). CC genotype associations were observed for LVH on echocardiography (*q* = 0.027), combined LVH (*q* = 0.028) and cardiac TOD (*q* = 0.039), and lower odds of carotid artery stenosis (*q* = 0.039). The C allele was associated with ≥ 2 damaged organ systems (*q* = 0.026).

**Conclusion:**

Preliminary findings suggest a possible involvement of the *MMP2* rs243865 polymorphism in target organ damage among patients with difficult-to-control hypertension.

## Introduction

Hypertension is the leading cause of morbidity and mortality worldwide (Mills, Stefanescu & He, 2020; Zhou et al., 2021). Despite the availability of numerous antihypertensive medications, blood pressure (BP) control rates remain low (Poulter et al., 2020). The prevalence of uncontrolled hypertension decreased slightly from 29% in 2010 to 26% in 2019, yet this remains above the target of 21%. This issue is particularly prevalent in Asian countries (Kario et al., 2024). In Vietnam, the prevalence of hypertension is rapidly increasing, whereas the proportions of hypertensives aware, treated and controlled were unacceptably low (Minh et al., 2021; Son et al., 2012). Beyond resistant hypertension, which has a well-defined clinical criterion, difficult-to-control hypertension represents a related but distinct entity. It describes cases in which blood pressure remains uncontrolled despite the use of maximally tolerated antihypertensive therapy, yet the condition does not fully meet the criteria for resistant hypertension. Although no standardized definition exists, it is commonly described as uncontrolled blood pressure after at least three months of treatment with three antihypertensive classes (including a diuretic), where optimal dosing cannot be achieved for various reasons (Oliveras & Schmieder, 2013). Difficult-to-control hypertension is associated with a high cardiovascular risk factor (Daugherty et al., 2012) and with increased all-cause mortality, cardiovascular mortality, and a higher prevalence of TOD compared to patients with controlled hypertension or normotensive individuals (Cuspidi et al., 2001; De la Sierra et al., 2012).

In the pathogenesis of hypertension, beyond hemodynamic and environmental factors (Oliveras & Schmieder, 2013; Papademetriou et al., 2011), genetic determinants have increasingly been recognized to play a significant role (Teixeira, Pereira & Krieger, 2018). Among these, the family of matrix metalloproteinase (MMPs) has been implicated in the risk of hypertension and uncontrolled hypertension (Bisogni et al., 2020; Qi et al., 2014; Sabbatini et al., 2017), primarily through mechanisms involvingvascular remodeling and TOD (Ritter et al., 2018). MMP2, one of the most extensively investigated enzymes in the matrix metalloproteinase family, plays a pivotal role in extracellular matrix turnover, as well as vascular and myocardial remodeling, and is closely linked to target organ damage and cardiovascular dysfunction in hypertension (Bisogni et al., 2020; Janicki et al., 2004).

Genetic polymorphisms within the MMP2 gene may modulate these pathophysiological processes, influencing susceptibility to cardiovascular events and target organ injury (Bisogni et al., 2020; Buraczynska et al., 2015). Among these variants, the rs243865 (−1306C>T) variant in the promoter region disrupts the Sp1 transcription factor binding site, thereby modifying transcriptional activity and MMP2 expression levels (Price, Greaves & Watkins, 2001). This variant has been identified as a potential risk factor for hypertension and TOD (Lacchini et al., 2012). However, studies investigating rs243865 in patients with uncontrolled hypertension remain limited, and the available evidence is still inconclusive (Ritter et al., 2018; Sabbatini et al., 2017).

In Vietnam, previous hypertension studies have primarily focused on clinical and biochemical factors, with limited investigation into genetic determinants. Recent research has explored genes within the cytochrome P450 and renin–angiotensin–aldosterone systems (Tran et al., 2024a; Tran et al., 2024b; Tran et al., 2021). However, studies on the MMP gene family—crucial for vascular remodeling and the progression of hypertension—remain scarce. This knowledge gap underscores the need to clarify the role of MMP2 in the pathogenesis of hypertension, particularly in patients with uncontrolled blood pressure. Therefore, the present study aimed to characterize the MMP2 rs243865 polymorphism and assess its association with target organ damage in this population.

## Materials & Methods

### Study design and population

**Figure 1:**
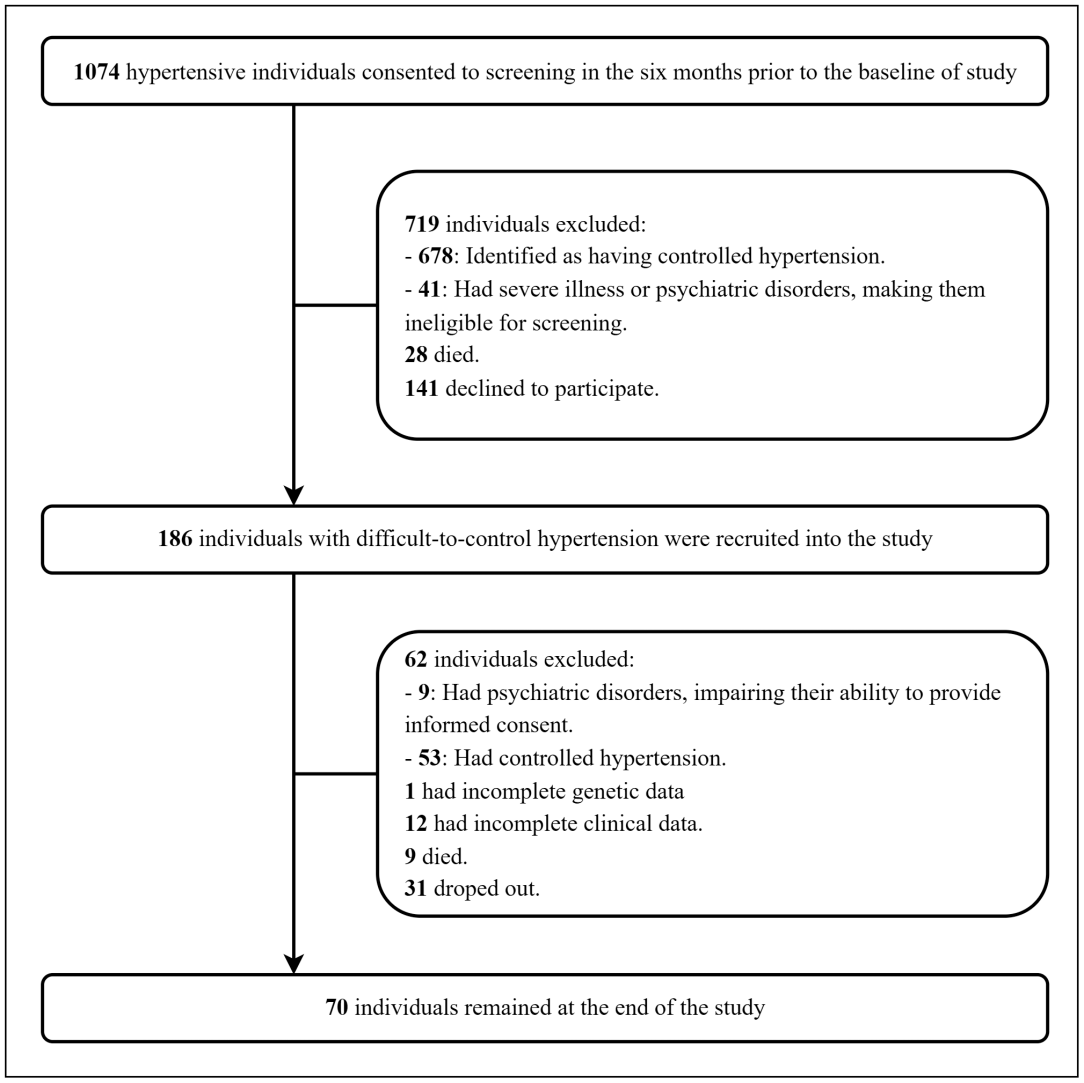
Flowchart of study participants.

This double-center cross-sectional study was conducted on adults with difficult-to-control hypertension, recruited from patients receiving care at Can Tho University of Medicine and Pharmacy Hospital and Can Tho Central General Hospital—two major medical centers in Can Tho, the economic, cultural, and healthcare hub of the Mekong Delta region, Vietnam. The study was carried out from April 2023 to June 2024. The overall study design and participant flow are shown in Fig. 1.

A non-probability convenience sampling approach was performed. Participants were mutually unrelated adults of Vietnamese ancestry aged 18 years or older who were diagnosed with difficult-to-control hypertension according to two criteria: (1) Patients whose blood pressure remained uncontrolled despite the use of at least three classes of antihypertensive drugs, including a diuretic (such as an angiotensin-converting enzyme inhibitor (ACEI) or an angiotensin II receptor blocker (ARB) in combination with a calcium channel blocker and a thiazide diuretic) for at least three months, yet did not reach the optimal or maximum dose for any reason, were classified as having difficult-to-control hypertension (Oliveras & Schmieder, 2013). (2) Failure to achieve a clinical systolic blood pressure below 140 mmHg and/or a diastolic blood pressure below 90 mmHg. Strict exclusion criteria were applied to ensure patient safety and maintain homogeneity within the study population. Patients requiring intensive care or those with severe comorbid conditions (such as sepsis or advanced cancer) were excluded. Additionally, individuals with a history of autoimmune diseases, those receiving immunosuppressive therapy, and patients diagnosed with dementia by a specialist were deemed ineligible for participation.

The sample size was determined using the formula $\mathrm{n}={\mathrm{Z}}_{1- \frac{\alpha }{2} }^{2} \frac{\mathrm{p} \left( 1-\mathrm{p} \right) }{{\mathrm{d}}^{2}} $. The calculation was based on a 95% confidence level and an 8% margin of error. The prevalence of the C allele in the *MMP2* rs243865 polymorphism was referenced from the previous study by Sabbatini et al. (2017), which reported a frequency of 87% in patients with resistant hypertension. Accordingly, the minimum required sample size to ensure statistical accuracy was 68 participants. In practice, we successfully enrolled 70 patients with difficult-to-control hypertension.

The study was approved by the Ethics Committee of Can Tho University of Medicine and Pharmacy (approval number: 23.046.HV-ÐHYDCT). Participation required voluntary informed consent, with each participant signing a written consent form at the time of enrollment. The study strictly adhered to the ethical principles outlined in the Declaration of Helsinki.

### Data collection

Anthropometric and clinical parameters included age, sex, height (to nearest 0.1 cm), weight (to nearest 0.1 kg), body mass index (BMI, kg/m^2^), smoking status, hypertension grade, dyslipidemia, type 2 diabetes mellitus, and history of stroke or transient ischemic attack (TIA). BMI was classified according to World Health Organization criteria for the Asian population (Anuurad et al., 2003). Participants were classified as smoking if they had smoked at least 100 cigarettes over their lifetime and were smoking at assessment (Yun et al., 2012). Hypertension grade was determined according to the International Society of Hypertension criteria 2020 (2020) (Unger et al., 2020). Dyslipidemia was diagnosed based on the National Cholesterol Education Program Adult Treatment Panel III criteria (2001) or current lipid-lowering therapy (Expert Panel on Detection Evaluation Treatment of High Blood Cholesterol in Adults, 2001). Diabetes was identified per American Diabetes Association criteria (2023) or ongoing glucose-lowering medication (ElSayed et al., 2023). A history of stroke or TIA was documented based on previous hospital admissions meeting the diagnostic criteria.

Target organ damage was defined as the presence of evidence of cardiac, renal, or vascular injury at enrollment, and as lesions that had been present for at least 1 year, as indicated by a review of prior medical records. All diagnostic procedures adhered to standardized protocols. Left ventricular hypertrophy (LVH) was defined by echocardiography as a left ventricular mass index >95 g/m^2^ (women) or >115 g/m^2^ (men) per European Society of Cardiology guidelines (2018). LVH on electrocardiography (ECG) was diagnosedby Sokolov-Lyon index (SV1 + RV5/V6 >35 mm), RaVL ≥ 11 mm, Cornell voltage-duration product (>2,440 mm ms), or Cornell voltage index (RaVL + SV3 >28 mm in men, >20 mm in women) (Williams et al., 2018). LVH was also confirmed when present on echocardiography or ECG. Myocardial ischemia on echocardiography was identified by abnormal systolic wall motion (hypokinesia <30%, akinesia <10%, dyskinesia with paradoxical outward motion) (Pislaru et al., 2001). ECG criteria for ischemia included pathological Q waves (≥ 40 ms width) or ST-segment depression (≥ 0.5 mm horizontal/downsloping) in two contiguous leads, excluding secondary changes from LVH or conduction blocks (Williams et al., 2018). Myocardial ischemia was also confirmed when present on echocardiography or ECG, and cardiac target organ damage was defined as the presence of at least one of LVH or myocardial ischemia. Decreased glomerular filtration rate was defined as an estimated glomerular filtration rate (eGFR) <60 mL/min/1.73 m^2^ using the Chronic Kidney Disease Epidemiology Collaboration (CKD-EPI) equation (2009) per Kidney Disease: Improving Global Outcomes (KDIGO) 2012 guidelines (KDIGO, 2013). Microalbuminuria was diagnosed by an albumin-to-creatinine ratio (ACR) ≥ 30 mg/g from 24-hour urine, and renal target organ damage was defined when microalbuminuria and or eGFR <60 mL/min/1.73 m^2^ were present. Peripheral vascular disease was identified by ankle-brachial index (ABI) >1.3 or <0.9, or arterial stenosis confirmed by peripheral Doppler ultrasound. Carotid stenosis was diagnosed when Doppler ultrasound showed focal arterial thickening ≥ 50% greater than adjacent intima-media thickness or focal intima-media thickness >1.5 mm protruding into the lumen (Johri et al., 2020). The number of target organs involved reflects the number of major organs (heart, kidneys, and blood vessels) that have sustained damage.

### Analysis of the *MMP2* rs243865 polymorphism

Peripheral venous blood samples were collected into two mL EDTA-coated tubes and immediately stored at −80 °C until further processing. Genomic DNA was extracted from whole blood using the GeneJET Genomic DNA Purification Kit (Thermo Scientific, Waltham, MA, USA), following the manufacturer’s protocol. The final elution volume of the genomic DNA solution was 200 µL. DNA concentration and purity were evaluated using a NanoDrop 2000 spectrophotometer (Thermo Scientific, Waltham, MA, USA), and samples with an optical density ratio (OD260/OD280) between 1.8 and 2.0 were considered of acceptable purity and stored at −20 °C for subsequent analyses. The MMP2 promoter polymorphism rs243865 (–1306 C>T) was genotyped using polymerase chain reaction (PCR). The amplification targeted a 1,007 bp fragment of the MMP2 gene using specific primers designed as follows: forward primer 5′-GACAAGCCTGAACTTGTCTG-3′and reverse primer 5′-TAGAGACGTTGGAACCAGAG-3′. PCR reactions were prepared in a total volume of 15 µL, comprising 1.5 µL 10X PCR buffer, 1.5 µL 2.5 mM dNTPs, 0.75 µL of each primer (10 µM), 0.1 µL Takara Taq™ Hot Start DNA Polymerase (Takara Bio, Shiga, Japan), 1 µL of genomic DNA, and 9.4 µL nuclease-free water (H_2_O-DEPC). PCR amplification was performed under the following cycling conditions: initial denaturation at 98 °C for 3 min; 40 cycles of denaturation at 98 °C for 10 s, annealing at 58 °C for 20 s, and extension at 72 °C for 50 s; followed by a final elongation at 72 °C for 2 min. Amplification products were confirmed *via* gel electrophoresis on agarose gels and visualized using a GelDoc-It^®^ Imager (UVP, Upland, CA, USA).

PCR products were purified using ExoSAP-IT (Applied Biosystems, Foster City, CA, USA) and subjected to direct sequencing. Sanger sequencing was conducted using the BigDye^®^ Terminator v3.1 Cycle Sequencing Kit (Applied Biosystems, Foster City, CA, USA) in a 9.5 µL reaction volume containing 0.5 µL BigDye Terminator, 1.5 µL 5X sequencing buffer, two µL sequencing primer (1.6 µM), one µL purified PCR product, and 4.5 µL nuclease-free water. Sequencing cycling conditions included an initial denaturation at 96 °C for 1 min, followed by 25 cycles of 96 °C for 10 s, 50 °C for 5 s, and 60 °C for 4 min. Post-sequencing, products were purified *via* ethanol precipitation and resuspended for analysis. Sequencing was performed on an ABI 3500^®^ Genetic Analyzer (Applied Biosystems, Foster City, CA, USA), and sequences were aligned and analyzed against the MMP2 reference sequence (GenBank accession NG_008989.1) using SeqScape^®^ Software v2.7 (Applied Biosystems, Foster City, CA, USA). Quality control included the use of known positive genotype controls and blind duplicate samples, with 100% concordance achieved to ensure genotyping accuracy and reproducibilit. Genotyping quality was high, with an overall call rate of 99.5% (185/186).

### Data analysis

Data collected in this study were encoded and processed using R version 4.5.0 and RStudio 5, utilizing libraries including tidyverse, dplyr, table1, compareGroups, ggplot2, survival, survminer, pROC, HardyWeinberg, Bioconductor (available at http://www.R-project.org). Quality-control procedures were adapted from Nguyen et al. (2025). Before analysis, records were screened for completeness and internal consistency; observations with missing key fields or implausible values were removed. Potential outliers were flagged using frequency/diagnostic plots and simple range checks; data wrangling used standard dplyr verbs (*e.g.*, filter(), mutate()), with missingness handled *via* drop_na()/replace_na To assess data entry reliability, a ∼10% random audit (sample_n()) was performed.

Categorical variables were coded numerically for analysis and are presented as n (%) (summarized with the table1 framework). For continuous variables, distributional assumptions were evaluated using the Shapiro–Wilk test (shapiro.test) together with visual inspection (histograms and Q–Q plots). Depending on normality and variance homogeneity (Levene’s test when relevant), group comparisons used Student’s t test (t.test) for normal data with equal variances, Welch’s t test for unequal variances, or the Mann–Whitney U test (wilcox.test) for non-normal data. For categorical outcomes, comparisons employed the chi-square test (chisq.test) or Fisher’s exact test (fisher.test) when expected cell counts were small. Continuous data are reported as mean (SD) or median (IQR), as appropriate.

Allele frequencies were calculated as the ratio of each allele count to twice the total number of subjects, and genotype frequencies (CC, CT, TT) were obtained by direct counting. Their distributions were visualized using bar charts, and associations with target organ damage were analyzed using Forest plots and tables that summarized odds ratios (OR), 95% confidence intervals (CI), and *p*-values. An OR greater than 1 indicated an increased risk. Hardy–Weinberg equilibrium was assessed using the exact test (HWExact), with a mid *p*-value > 0.05 considered consistent with equilibrium. All analyses were two-tailed, with statistical significance set at *p* < 0.05.

Associations between genotype (CC *vs.* CT+TT) and each predefined outcome were examined using logistic regression, both univariable and multivariable models adjusted for age, sex, dyslipidemia, duration of hypertension, and duration of type 2 diabetes. Given the exploratory analysis with multiple hypotheses (*m* = 15), the false discovery rate (FDR) was controlled using q-values according to Storey’s method, implemented in the qvalue package (Krzywinski & Altman, 2014; Storey & Tibshirani, 2003). Only outcomes analyzed *via* logistic regression (excluding those assessed by Fisher’s exact test due to separation) were included in FDR correction. Final inferences were based on *q* < 0.05, with *p*-values provided as supplementary information.

## Results

A total of 70 patients with difficult-to-control hypertension were enrolled in the study and followed until its completion. Regarding general characteristics, women were predominant, with a women-to-men ratio of approximately 1.6. The majority of participants were elderly, with a mean age of 66.47 ± 14.77 years, and had a normal body weight (70.0%). In terms of lifestyle habits, approximately one-third of the participants were smokers. Regarding medical history, about one-third had dyslipidemia, one-quarter had concomitant type 2 diabetes mellitus, and one-fifth had a history of stroke or TIA. Notably, most participants had stage II hypertension (87.1%). The detailed information is presented in Table 1.

**Table 1:** Baseline characteristics of 70 participants.

Characteristics		Frequency (n)	Percentage (%)
Gender			
Men		27	38.6
Women		43	61.4
Age (years)	Mean ± SD	66.47 ± 14.77	
≥ 65		41	58.6
<65		29	41.4
BMI (kg/m^2^)	Mean ± SD	23.75 ± 3.18	
Underweight (<18.5 kg/m^2^)		1	1.4
Normal weight (18.5-<23 kg/m^2^)		49	70.0
Overweight - Obesity (≥ 23 kg/m^2^)		20	28.6
Smoking			
Yes		22	31.4
No		48	68.6
Hypertension			
Grade I (140–159 mmHg)		9	12.9
Grade II (≥ 160 mmHg)		61	87.1
Diabetes mellitus			
Yes		18	25.7
No		52	74.3
Dyslipidemia			
Yes		23	32.9
No		47	67.1
Stroke or TIA			
Yes		14	20.0
No		56	80.0

**Notes.**

BMI body mass index SD standard deviation TIA transient ischemic attack

**Figure 2:**
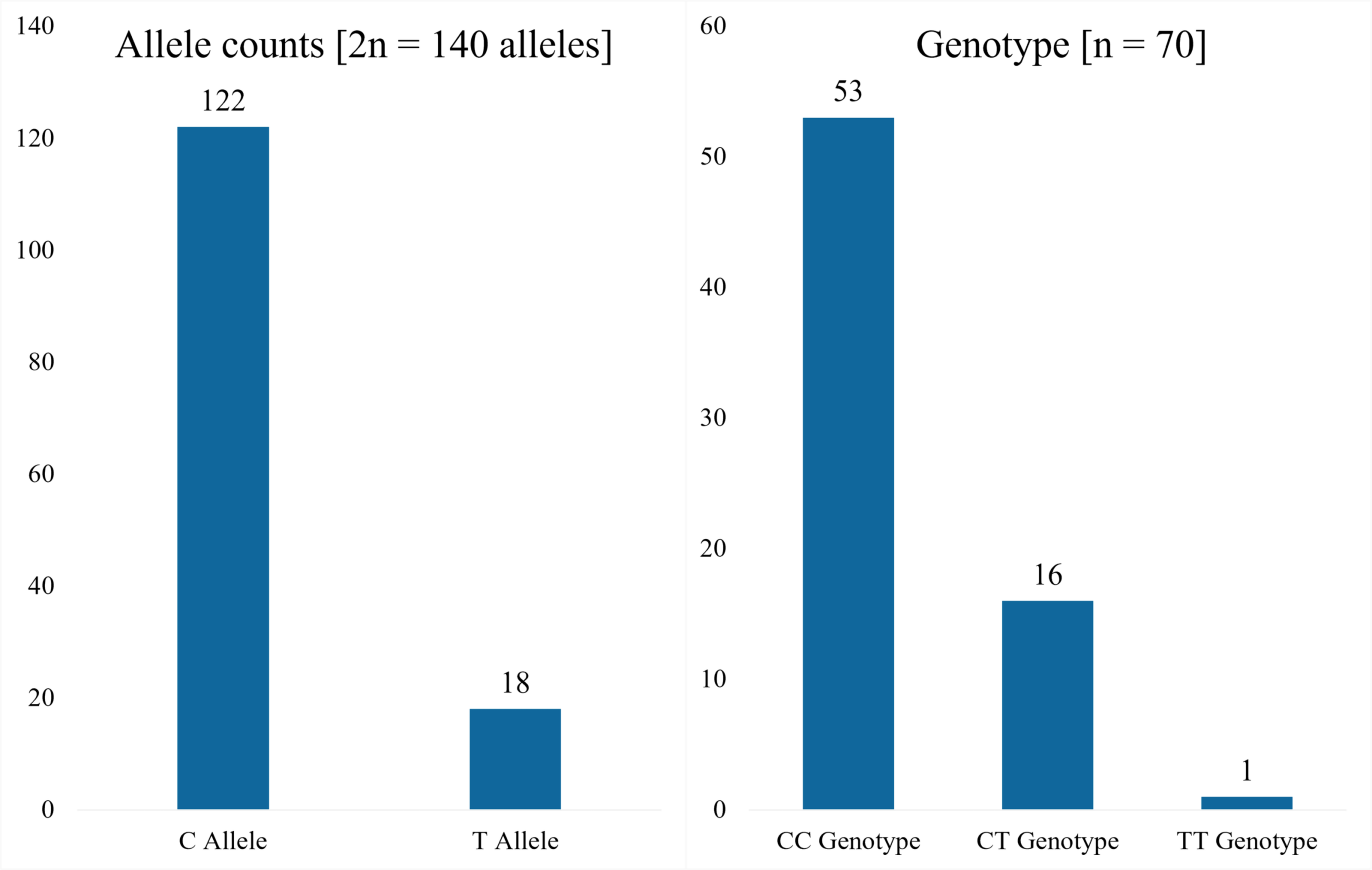
The rs243865 polymorphism of the *MMP2* gene in difficulty-to-control hypertensive patients.

Analysis of the rs243865 polymorphism in the *MMP2* gene among 70 participants revealed that the majority carried the C allele, accounting for approximately seven-eighths of the total. Additionally, the CC genotype was the most prevalent, observed in three-quarters of the participants, while the remainder were mostly of the CT genotype. Only one case of the TT genotype was identified (Fig. 2). An exact HWE test (HWExact) indicated no deviation from equilibrium (mid-p = 0.87).

Regarding allele distribution, the C allele group exhibited a higher prevalence of dyslipidemia compared to the T allele group (36.1% *vs.* 11.1%, *p* = 0.035). However, no statistically significant differences were observed in allele distribution concerning other characteristics, including sex, overweight/obesity status, severity of hypertension, type 2 diabetes mellitus, and history of stroke or transient ischemic attack (all *p* > 0.05). The detailed information is presented in Table 2.

**Table 2:** Distribution of alleles according to the baseline characteristics of the study subjects. The proportions of gender, overweight-obesity, hypertension, diabetes mellitus, dyslipidemia, and stroke or TIA are expressed as the number of alleles along with their percentage given in parentheses with respect to the total number of alleles in each group (C allele and T allele). The differences are compared according to the a Pearson Chi-Square or b Fisher’s Exact Test. Statistical significance: *p* < 0.05.

Characteristics	Overall allele count (*n* = 140)	Allele C (*n* = 122)	Allele T (*n* = 18)	*p*-value
Gender				0.976^a^
Men	54 (38.6)	47 (38.5)	7 (38.9)	
Women	86 (61.4)	75 (61.5)	11 (61.1)	
Overweight-Obesity				0.231^a^
Yes	40 (28.6)	37 (30.3)	3 (16.7)	
No	100 (71.4)	85 (69.7)	15 (83.3)	
Hypertension				0.704^b^
Grade I (140–159 mmHg)	18 (12.9)	15 (12.3)	3 (16.7)	
Grade II (≥ 160 mmHg)	122 (87.1)	107 (87.7)	15 (83.3)	
Diabetes mellitus				1^b^
Yes	36 (25.7)	32 (26.2)	4 (22.2)	
No	104 (74.3)	90 (73.8)	14 (77.8)	
Dyslipidemia				**0.035** ^a^
Yes	46 (32.9)	44 (36.1)	2 (11.1)	
No	94 (67.1)	78 (63.9)	16 (88.9)	
Stroke or TIA				1^b^
Yes	28 (20)	25 (20.5)	3 (16.7)	
No	112 (80)	97 (79.5)	15 (83.3)	

**Notes.**

BMI body mass index SD standard deviation TIA transient ischemic attack

Bold values indicate statistical significance (*p* < 0.05).

Table 3 showed that the CC genotype exhibited a higher prevalence of dyslipidemia compared to the CT-TT genotype (26.4% *vs.* 23.5%, *p* = 0.033). However, no statistically significant differences were observed in genotype distribution concerning other characteristics, including sex, overweight/obesity status, severity of hypertension, type 2 diabetes mellitus, and history of stroke or transient ischemic attack (all *p* > 0.05).

**Table 3:** Distribution of genotypes according to the baseline characteristics of the study subjects (*n* = 70). The proportions of gender, overweight-obesity, hypertension, diabetes mellitus, dyslipidemia, and stroke or TIA are expressed as the number of genotypes along with their percentage (given in parentheses), with respect to the total number of genotypes in each group (CC genotype and CT-TT genotype). The differences are compared according to the a Pearson Chi-Square or b Fisher’s Exact Test.

Characteristics n (%)	Total (*n* = 70)	Genotype CC (*n* = 53)	Genotype CT (*n* = 16)	Genotype TT (*n* = 1)	*p*-value*
Gender					0.750^a^
Men	27 (38.6)	21 (39.6)	5 (31.2)	1 (100)	
Women	43 (61.4)	32 (60.4)	11 (68.8)	0 (0.0)	
Overweight - Obesity					0.359^b^
Yes	50 (71.4)	36 (67.9)	13 (81.3)	0 (0.0)	
No	20 (28.6)	17 (32.1)	3 (18.8)	1 (100)	
Hypertension					0.678^b^
Grade I (140–159 mmHg)	9 (12.9)	6 (11.3)	3 (18.8)	1 (100)	
Grade II (≥ 160 mmHg)	61 (87.1)	47 (88.7)	13 (81.2)	0 (0.0)	
Diabetes mellitus					1^b^
Yes	18 (25.7)	14 (26.4)	4 (25.0)	0 (0.0)	
No	52 (74.3)	39 (73.6)	12 (75.0)	1 (100)	
Dyslipidemia					**0.033** ^a^
Yes	23 (32.9)	21 (39.6)	2 (12.5)	0 (0.0)	
No	47 (67.1)	32 (60.4)	14 (87.5)	1 (100)	
Stroke or TIA					0.492^b^
Yes	14 (20.0)	12 (22.6)	1 (6.2)	1 (100)	
No	56 (80.0)	41 (77.4)	15 (93.8)	0 (0.0)	

**Notes.**

* *p*-value calculated under the T-allele dominant model (CT + TT *vs* CC). Statistical significance (bold values): *p* < 0.05.

BMI body mass index SD standard deviation TIA transient ischemic attack

**Figure 3:**
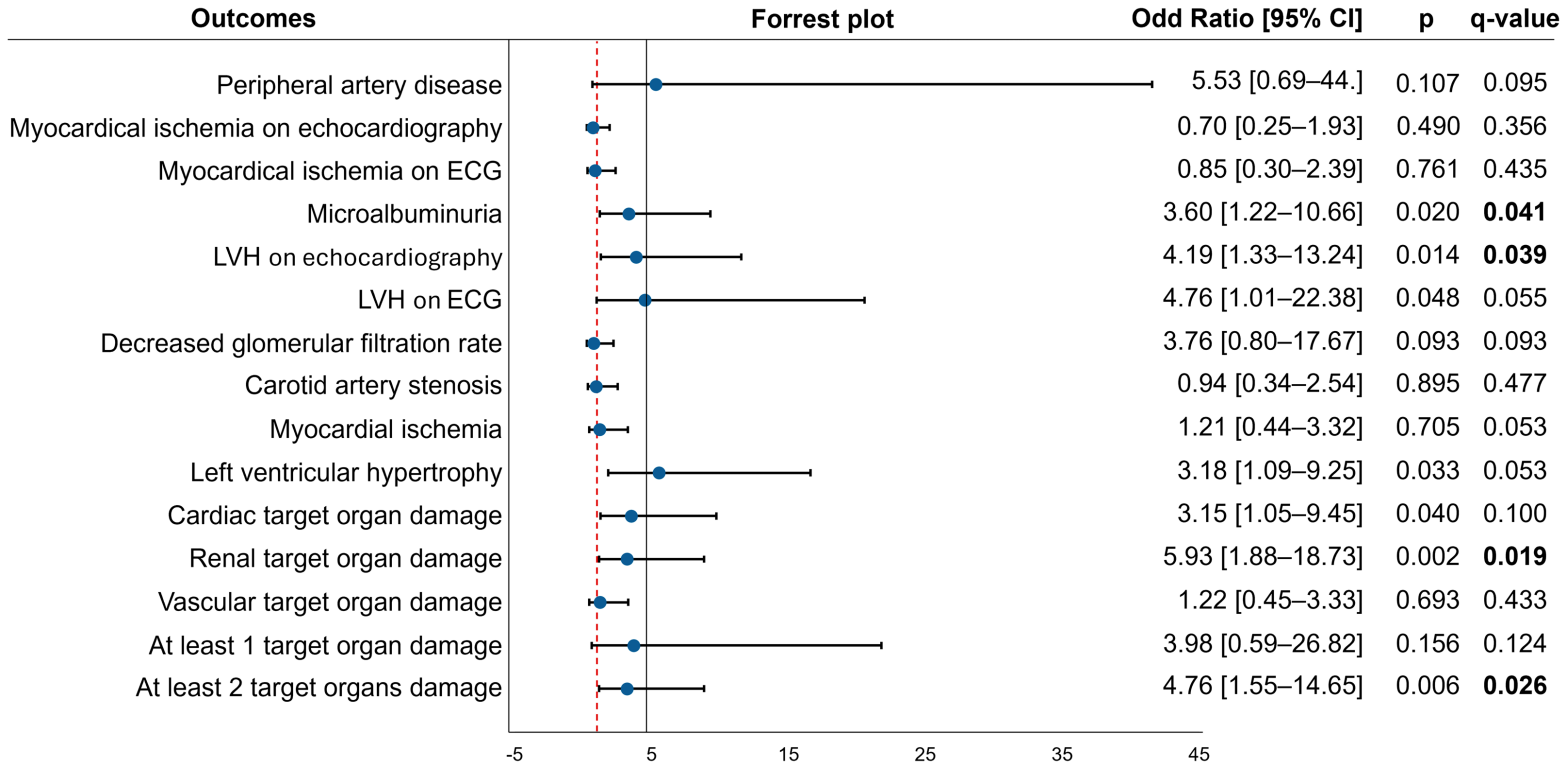
The association between the alleles of the MMP2 rs243865 polymorphism and target organ damage.

In patients with difficult-to-control hypertension, the C allele was significantly associated with four types of target organ damage after FDR correction: microalbuminuria (OR = 3.60; 95% CI [1.22–10.66]; *q* = 0.041), LVH on echocardiography (OR = 4.19; 95% CI [1.33–13.24]; *q* = 0.039), renal involvement (OR = 5.93; 95% CI [1.88–18.73]; *q* = 0.019), and multi-organ damage affecting two or more organs (OR = 4.76; 95% CI [1.55–14.65]; *q* = 0.026) (Fig. 3).

Among the 70 patients, under a dominant model for the T allele (CC *vs.* CT + TT) and after FDR correction the CC genotype was associated with higher odds of several target organ damage indices: microalbuminuria (OR = 3.58; 95% CI [0.99–12.95]; *q* = 0.049), LVH on echocardiography (OR = 6.62; 95% CI [1.62–27.05]; *q* = 0.027), LVH on ECG (OR = 5.10; 95% CI [0.95–27.37]; *q* = 0.049), and combined LVH (OR = 5.42; 95% CI [1.40–20.94]; *q* = 0.028). The CC genotype also showed higher odds of cardiac damage (OR = 4.45; 95% CI [1.13–17.52]; *q* = 0.039) and renal damage (OR = 6.07; 95% CI [1.56–23.56]; *q* = 0.027). Conversely, the CC genotype was associated with lower odds of carotid artery stenosis (OR = 0.18; 95% CI [0.04–0.85]; *q* = 0.039) (Table 4).

## Discussion

This is one of the few studies in Vietnam investigating the matrix metalloproteinase enzyme family. The study focuses on characterizing the single nucleotide polymorphism rs243865 of the *MMP2* gene and determining its association with target organ damage in patients with difficult-to-control hypertension. Among the study participants, 75.7% carried the homozygous CC genotype, and the C allele was predominant with a frequency of 87.1%. The genotype distribution conformed to the Hardy–Weinberg equilibrium (mid-p = 0.87). The C allele and CC genotype were significantly associated with multiple target organ damages. These findings suggest that the rs243865 polymorphism may contribute to the genetic susceptibility to cardio-renal complications in patients with difficult-to-control hypertension.

**Table 4:** The association between the genotypes of the MMP2 rs243865 polymorphism and target organ damage (*n* = 70).

**Outcomes**	**CC** ***n* = 53**	**CT** ***n* = 16**	**TT** ***n* = 1**	**OR (95% CI)** ^a^ ^,^ ^b^	**p-value** ^b^	**q-value** ^b^	**OR (95% CI)** ^a^ ^,^ ^c^	**p-value** ^c^	**q-value** ^c^
Peripheral artery disease	14 (26.4)	1 (6.2)	0 (0.0)	5.74 (0.70–47.40)	0.105	0.164	2.81 (0.29–27.20)	0.373	0.203
Myocardial ischemia on echocardiography	19 (35.8)	6 (37.5)	1 (100)	0.80 (0.26–2.44)	0.692	0.755	1.11 (0.32–3.93)	0.867	0.346
Myocardial ischemia on ECG	19 (35.8)	5 (31.2)	1 (100)	1.02 (0.33–3.21)	0.966	0.913	0.76 (0.20–2.93)	0.695	0.321
Microalbuminuria	38 (71.7)	7 (43.8)	0 (0.0)	3.62 (1.16–11.27)	0.026	0.062	3.58 (0.99–12.95)	0.051	**0.049**
Left ventricular hypertrophy on echocardiography	34 (64.2)	5 (31.2)	0 (0.0)	4.29 (1.31–14.04)	0.015	**0.045**	6.62 (1.62–27.05)	0.008	**0.027**
Left ventricular hypertrophy on ECG	21 (39.6)	2 (12.5)	0 (0.0)	4.92 (1.02–23.77)	0.047	0.095	5.10 (0.95–27.37)	0.057	**0.049**
Decreased glomerular filtration rate	18 (34.0)	2 (12.5)	0 (0.0)	3.86 (0.79–18.75)	0.094	0.164	3.04 (0.56–16.43	0.196	0.117
Carotid artery stenosis	25 (47.2)	9 (56.2)	0 (0.0)	0.79 (0.27–2.37)	0.679	0.755	0.18 (0.04–0.85)	0.031	**0.039**
Myocardial ischemia	27 (50.9)	6 (37.5)	1 (100)	1.48 (0.49–4.48)	0.484	0.624	1.62 (0.47–5.60)	0.443	0.221
Left ventricular hypertrophy	40 (75.5)	6 (37.5)	1 (100)	4.40 (1.39–13.89)	0.011	**0.045**	5.42 (1.40–20.94	0.014	**0.028**
Cardiac target organ damage	44 (83.0)	8 (50.0)	1 (100)	4.35 (1.32–14.31)	0.015	**0.045**	4.45 (1.13–17.52)	0.032	**0.039**
Renal target organ damage	43 (81.1)	7 (43.8)	0 (0.0)	6.14 (1.88–20.11)	0.003	**0.038**	6.07 (1.56–23.56)	0.009	**0.027**
Vascular target organ damage	29 (54.7)	9 (56.2)	0 (0.0)	1.07 (0.36–3.21)	0.898	0.909	0.33 (0.07–1.50)	0.151	0.113
≥ 1 target organ damage	52 (98.1)	14 (87.5)	1 (100)	6.93 (0.59–81.82)	0.124	0.175	0.69 (0.03–18.40)	0.825	0.346
≥ 2 target organs damage	43 (81.1)	8 (50.0)	0 (0.0)	4.84 (1.49–15.66)	0.008	**0.045**	2.50 (0.66–9.52)	0.178	0.117

**Notes.**

a Calculated under the T-allele dominant model (CC *vs* CT+TT).

b Crude.

c Adjusted for age, sex, dislipidemia, duration of hypertension and duration of diabetes.

ECG electrocardiograph

Bold values indicate statistical significance (*p* < 0.05).

In recent years, members of the gelatinase subgroup within the MMP family, particularly MMP2, have been identified as important contributors to hypertension due to their involvement in vascular endothelial degradation and remodeling (Bisogni et al., 2020). The *MMP2* gene, located on chromosome 16, contains regulatory region variants that may influence the transcriptional activity of adjacent genes (Buraczynska et al., 2015), suggesting a potential link between *MMP2* polymorphisms, circulating MMP2 levels, and the development of hypertension. Our findings are consistent with previous reports. In China, Qi et al. (2014) reported higher frequencies of the CC genotype (57.5% *vs.* 51.7%, *p* = 0.044) and the C allele (75.2% *vs.* 71.4%, *p* = 0.011) in hypertensive patients compared with controls (Qi et al., 2014). In Brazil, Sabbatini et al. (2017) found a higher prevalence of the CC genotype in patients with resistant hypertension (81% *vs* 68%), which was significantly associated with increased odds of resistant hypertension (Sabbatini et al., 2017). These findings suggest that the MMP2 rs243865 C allele and CC genotype may increase susceptibility to hypertension and its resistant form. However, results remain inconsistent, as no significant associations were observed in Caucasian populations (Moskalenko et al., 2021). Similarly, Qi et al. (2014) further demonstrated that rs243865 was not directly associated with hypertension but exhibited a significant interaction with MMP3, forming the strongest predictive indicator for the disease (Qi et al., 2014). This highlights the importance of gene–gene and gene–environment interactions, as the effect of a single variant may be masked without accounting for other loci or environmental influences (Phillips, 2008; Soremekun et al., 2023; Kato, 2012). Minimizing genetic heterogeneity through the inclusion of homogeneous populations and controlling factors such as diet, physical activity, and access to healthcare is crucial for enhancing the reliability of association studies.

Clinically, *MMP2* is implicated in hypertensive target organ damage, particularly in the development of left ventricular hypertrophy. In this study, the MMP2 rs243865 C allele and CC genotype were associated with increased odds of LVH, as identified by both electrocardiography and echocardiography, likely reflecting MMP2′s role in collagen degradation and myocardial remodeling that promote ventricular hypertrophy and dilation (Bisogni et al., 2020; Janicki et al., 2004; Li et al., 2023). However, the findings across populations remain inconsistent. Lacchini et al. (2012) reported a protective effect of the CC genotype, characterized by a smaller left ventricular end-diastolic diameter and lower left ventricular mass index (Lacchini et al., 2012). Conversely, Ritter et al. (2018) observed a higher prevalence of LVH in CT and TT carriers, but the association lost significance after adjustment (Ritter et al., 2018). Mechanistically, the rs243865 variant disrupts the binding site of the Sp1 transcription factor, resulting in approximately 1.6-fold higher transcriptional activity of *MMP2* in C allele carriers (Price, Greaves & Watkins, 2001). In ventricular hypertrophy, enhanced gelatinase activity facilitates LVH progression (Janicki et al., 2004). Therefore, the rs243865 polymorphism may exert complex effects on myocardial remodeling, and interstudy discrepancies likely reflect interactions between genetic background, environmental exposures, and molecular signaling diversity.

Our findings also indicated a lower detection rate of carotid atherosclerosis among individuals with the CC genotype compared with T allele carriers. However, this does not suggest a protective effect. Volcik et al. (2010) reported that CC genotype carriers had fibrous caps approximately 0.1 mm thinner than those of T allele carriers, with no difference in lipid core size (Volcik et al., 2010), implying that unstable plaques may be less detectable by ultrasound (Takaya et al., 2006; Virmani et al., 2006). Thus, the lower detection frequency could reflect a more unstable plaque phenotype and potentially greater cardiovascular risk. Further studies employing advanced imaging and detailed histopathological assessments are warranted to validate this observation.

We also found that microalbuminuria and hypertension-related renal damage were associated with the C allele and CC genotype, contrasting with the findings of Sabbatini et al. (2017) and Ritter et al. (2018), who reported no significant association between this polymorphism and microalbuminuria (Ritter et al., 2018; Sabbatini et al., 2017). Previous studies have demonstrated that increased MMP2 and MMP9 activity correlates with hypertension-induced renal dysfunction, even in moderate disease stages (Rodríguez-Sánchez et al., 2019). These results provide additional evidence linking rs243865 to renal injury, such as microalbuminuria. Mechanistically, elevated MMP2 activity may degrade type IV collagen and other components of the glomerular basement membrane, compromising the filtration barrier and leading to proteinuria (Wolosowicz, Prokopiuk & Kaminski, 2024). Nevertheless, given the limited available evidence, further studies are warranted to clarify the genetic and environmental determinants of *MMP2* regulation and their clinical relevance.

Notably, CC carriers showed a higher likelihood of multi-organ involvement than T allele carriers, highlighting the systemic genetic contribution to difficult-to-control hypertension. This pattern is consistent with evidence that resistant hypertension is associated with greater heart, kidney, and vascular injury compared with well-controlled cases (Cuspidi et al., 2001; De la Sierra et al., 2012). Genetically, MMP family variants—particularly MMP2 rs243865—have been linked to an increased risk of hypertension and adverse cardiovascular phenotypes (Qi et al., 2014). Furthermore, rs243865 and rs243866 have been associated with blood pressure control and organ damage in cohorts of resistant hypertension patients (Ritter et al., 2018).

This study employed a rigorous sampling approach and a reproducible methodology, demonstrating an association between the MMP2 −1306C/T (rs243865) polymorphism and difficult-to-control hypertension. However, several limitations should be acknowledged. The relatively small convenience sample (*n* = 70) from two centers may have introduced selection bias, reduced statistical power, and limited generalizability. The cross-sectional design, lacking a well-controlled hypertensive comparison group precludes causal inference. Residual confounding related to lifestyle, treatment regimens, and medication adherence may also persist. Additionally, due to resource constraints, circulating MMP2 levels and functional biomarkers were not assessed. Future studies with larger cohorts, broader genotyping, comprehensive clinical characterization, and longitudinal biomarker assessment are warranted to confirm and elucidate these associations.

## Conclusions

In summary, this study provides preliminary evidence suggesting a possible association between the *MMP2* rs243865 polymorphism and target organ damage in patients with difficult-to-control hypertension. However, these findings are exploratory and warrant further confirmatory studies.

##  Supplemental Information

10.7717/peerj.20489/supp-1 Supplemental Information 1 GenBank Accession Numbers for NOS3 rs243865 Sequences

10.7717/peerj.20489/supp-2 Supplemental Information 2 STROBE Checklist for Cross Sectional study

10.7717/peerj.20489/supp-3 Supplemental Information 3 Raw data

10.7717/peerj.20489/supp-4 Supplemental Information 4 Comparison of two studies

## Additional Information and Declarations

### Data Availability

The following information was supplied regarding data availability: The raw data are available in the Supplemental Files. The raw sequence data are available at DDBJ: LC909676, LC909677, LC909678.
