# Supplementary material for: Matrix metalloproteinase-2 (MMP2) rs243865 polymorphism and target end-organ damage in difficult-to-control hypertensive patients
Source: PeerJ. 2026 Mar 6;14:e20489. doi: 10.7717/peerj.20489 (PMC12970309; doi:10.7717/peerj.20489)
Supplement: Supplemental Information 1 [file peerj-14-20489-s001.docx]

Supplementary Table 1. GenBank Accession Numbers for NOS3 rs243865 Sequences

| Sample ID | Genotype | Accession Number |
| --- | --- | --- |
| DHYD18B10 | CC | PV640820 |
| DHYD23G10 | TT | PV640821 |
| DHYD29E11 | CT | PV640822 |

The nucleotide sequences of the NOS3 promoter region encompassing rs243865 (genotypes CC, CT, and TT) were deposited in the GenBank third-party database. Accession numbers and direct links to the records are provided in Supplementary Table 1.
