# Supplementary material for: Matrix metalloproteinase-2 (MMP2) rs243865 polymorphism and target end-organ damage in difficult-to-control hypertensive patients
Source: PeerJ. 2026 Mar 6;14:e20489. doi: 10.7717/peerj.20489 (PMC12970309; doi:10.7717/peerj.20489)
Supplement: Supplemental Information 4 [file peerj-14-20489-s004.docx]

|  | PMID: 40311090 | Our study |
| --- | --- | --- |
| Patient population | Resistant hypertension | Difficult-to-control hypertension |
| Sample size | 78 patients | 70 patients |
| Primary outcomes assessments | Identification of associations between MMP2 rs243865 polymorphism and:  Cardiac damage: assessed by left ventricular hypertrophy (via echocardiography using LVMI or by electrocardiography) and left ventricular ejection fraction.  Cerebral damage: neuroimaging, and documented history of stroke or transient ischemic attack.  Renal damage: albumin-to-creatinine ratio in urine.  Vascular damage: pulse wave velocity, ankle-brachial index, and carotid artery damage assessed by ultrasound with intima-media thickness ≥ 0.9 mm or ≥ 50% luminal stenosis. | Evaluated at both baseline and after one year of follow-up.  Identification of associations between MMP2 rs243865 polymorphism and:  Cardiac damage: defined by left ventricular hypertrophy (LVMI measured by echocardiography or ECG) and myocardial ischemia (detected by echocardiography or ECG).  Renal damage: estimated glomerular filtration rate, urinary albumin-to-creatinine ratio.  Vascular damage: ankle-brachial index, or carotid artery stenosis confirmed by Doppler ultrasound with ≥ 50% luminal narrowing or focal intima-media thickness > 1.5 mm protruding into the lumen. |
